# Successful repigmentation with ritlecitinib and combined home-based phototherapy in an intractable case of generalized vitiligo^☆^

**DOI:** 10.1016/j.abd.2025.501157

**Published:** 2025-07-14

**Authors:** Xiu-Kun Sun, Ai-E Xu

**Affiliations:** Department of Dermatology, Hangzhou Third People's Hospital, Hangzhou, China

Dear Editor,

Generalized vitiligo usually requires systemic treatment due to its large lesion areas. Systemic glucocorticoid and traditional immunosuppressants have a limited role due to their side effects. Recently, novel agents targeting Janus kinase (JAK), a family of tyrosine kinases that regulates cytokine signaling, have emerged. JAK inhibitors are a promising option for the treatment of vitiligo orally or by topical application.^1^ Ritlecitinib is an oral JAK inhibitor approved by the FDA for the treatment of severe alopecia areata. In a Phase 2b trial, ritlecitinib demonstrated significant improvement in the Facial Vitiligo Area Scoring Index in patients with active nonsegmental vitiligo.^2^

Here, we report a case with generalized vitiligo refractory to systemic and topical corticosteroids, which responded well to oral ritlecitinib combined with phototherapy by a 308 nm excimer lamp for home-based use.

The case is a 26-year-old man who has been suffering from vitiligo for 5 years. A small white patch inniciated on the abdomen, which then increased gradually. The patient began to take traditional herbs and apply tacrolimus 0.1% ointment on the affected areas, but the lesions were still progressing, and finally spread to the trunk, thighs, palms, and soles. In 2021, he visited our clinic. The patient was diagnosed with progressive vitiligo and started receiving betamethasone injections to control the disease combined with whole-body Narrow-Band Ultraviolet B (NB-UVB) phototherapy. Diprospan® (suspension of 7 mg compound betamethasone) was injected intramuscularly four times at an interval of 3‒4 weeks. The radiation source of the phototherapy was the Waldmann UV therapy system, which used Phillips TL-01 fluorescent lamps with a radiation spectrum of 310‒315 nm and a peak of 311 nm. The patient was exposed to NB-UVB therapy two times per week, on non-consecutive days for 45 sessions. However, these treatments showed no obvious effectiveness. Most areas of the trunk, thighs, palms and soles were involved by vitiligo. The treatment was changed to ritlecitinib 50 mg once a day, combined phototherapy with a handhold 308 nm excimer lamp every other day. Excimer light starting doses ranged from 180 mJ/cm^2^ to 360 mJ/cm^2^, depending on the body site, and post-treatment erythema-guided adjustments of subsequent doses. Approximately 2 months after that, the vitiligo of this patient was well controlled, with 80% repigmentation of the lesions at the trunk (Fig. 1) and thighs, while palms and soles remained with no obvious repigmentation. The patient received ritlecitinib orally for half a year and currently undergoing maintenance therapy at a dose of 50 mg every other day. No adverse effect including abnormality of biochemistry metabolism was found, showing ritlecitinib safety.

**Figure 1:**
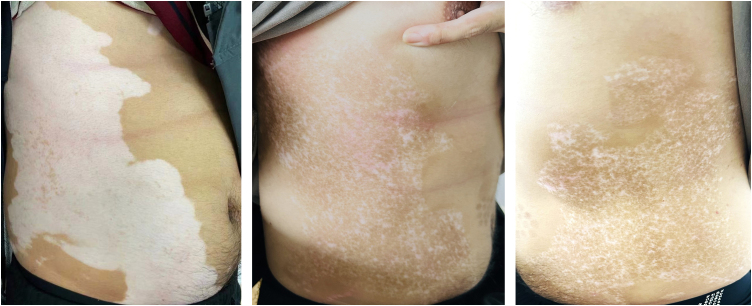
Right truncal lesions of vitiligo at baseline; repigmentation of bilateral truncal lesions after 2 months of ritlecitinib combined with 308 nm excimer light phototherapy.

Ritlecitinib, a JAK3/TEC inhibitor, shows therapeutic effects on alopecia areata by inhibiting IL-5 and IFN-γ (ritlecitinib may decrease the production of IFN-γ via an indirect mechanism, related to TEC kinase inhibition), which are also pathogenic cytokines in vitiligo and can therefore be used to treat this condition. A recent study revealed that ritlecitinib-treated vitiligo patients showed downregulation of immune biomarkers and upregulation of melanocyte-related markers.^3^

Given the encouraging results of the phase 2 clinical trial of ritlecitinib for the treatment of vitiligo, the patient accepted the off-label use this medicine after experiencing failures in previous treatments. The oral ritlecitinib therapy achieved great improvement within 2 months. Although the repigmentation was partly attributed to phototherapy, ritlecitinib played a key role in the success of this case, since the patient has already used phototherapy multiple times in the past with poor efficacy, and ideal therapeutic effects appeared only after oral administration of ritlecitinib. In a report, oral tofacitinib also could stop the progression of active vitiligo, when combined with phototherapy.^4^

Furthermore, ritlecitinib is highly selective for JAK3. The most common adverse events of ritlecitinib were nasopharyngitis, upper respiratory tract infection, and headache as observed in clinical trials for vitiligo^2^ and long-term treatment of alopecia areata.^5^ No adverse reactions were found in our patient, but it is still necessary to closely monitor the occurrence of side effects.

Finally, home phototherapy should be considered for its economic value, good tolerability, adherence, and efficacy.^6^ In this case, both oral medication and 308 nm excimer light phototherapy are performed at home, providing a convenient treatment option for the patient.

## Financial support

None declared.

## Authors' contributions

Xiu-Kun Sun: Contributed to the conception, design, and data analysis of this study.

Ai-E Xu: Contributed to the conception, design, and data analysis of this study.

## Conflicts of interest

None declared.
